# Animal Models of Human Pathology: Revision, Relevance and Refinements

**DOI:** 10.3390/biomedicines12112418

**Published:** 2024-10-22

**Authors:** Martina Perše

**Affiliations:** Institute of Pathology, Faculty of Medicine, University of Ljubljana, 1000 Ljubljana, Slovenia; martina.perse@mf.uni-lj.si

**Animal Models of Human Pathology**

The term “animal model” refers to a non-human species employed in biomedical research due to its capacity to replicate certain biological processes or diseases found in humans. This resemblance allows researchers to use findings from animal studies to understand human physiology and the underlying mechanisms of various diseases [1,2].

Animal models enable scientists to explore hypotheses in a controlled environment. Through these models, researchers can investigate the initiation and progression of diseases, evaluate potential therapeutic interventions, and study the effects of genetic manipulation and environmental factors on health outcomes [3].

To provide relevant, translatable scientific data, and to ensure the most beneficial use of animal models, animal research is conducted under controlled environmental conditions using animals whose genetic and microbiological backgrounds are known [4,5,6].

**Variables in Animal Research**

There are numerous examples of how a particular factor affects the study outcome, or even the characteristics, of an animal model. For instance, the C57BL/6J strain is a model for examining the multigenic factors involved in diet-induced obesity [7]. However, the extent of weight gain in this model can differ significantly across studies. In some instances, weight gain is minimal, while in others, it is substantial. This variability might lead one to view C57BL/6J mice as an inconsistent model for diet-induced obesity. Nevertheless, exploring the reasons behind these differing outcomes has revealed the significant roles of gut microbiota and thermoregulation [8] and in acclimating imported mice to the husbandry conditions of their new environment [9]. Emerging evidence shows that numerous factors, such as sex [10,11,12,13], genetic background [14,15,16], microbiological state [17,18,19], microbiota [20], environmental and physiological variables (housing conditions, food quality, enrichment, caging) [21,22,23], pain management [24,25], handling [26], humane and study endpoints [27], euthanasia criteria, training of personnel [28], etc., impact the study outcome. Importantly, all of these factors differ both among facilities and also in the same facility over time.

To help improve the reporting of factors involved in animal studies, and thus increase the quality and reproducibility of animal research, including animal models, various strategies have been proposed, including recommendations and guidelines such as the ARRIVE guidelines [29,30,31], Gold standard publication checklist [32], and PREPARE guidelines [33]. These guidelines highlight and list the basic factors that impact the physiological, immunological, or biochemical mechanisms in animals and influence the parameters under investigation, making them an essential part of every animal study.

**The Ideal Model Does Not Exist**

Historically, animal disease models were broadly divided into five groups: experimentally induced, genetically modified, spontaneous, orphan, and negative disease models. Experimentally induced disease models are created by inducing a disease state into a healthy animal through various means, such as, for instance, using chemical agents (chemically induced), surgical or physical interventions (mechanically induced), cell transplantation (xenograft, autograft, allograft), or biological agents like viruses or bacteria. Genetically modified disease models are those in which a disease created by the manipulation of genetic material (e.g., using genetic engineering techniques or carcinogens like ENU that induce point mutations) is transmitted to the next generation. In a spontaneous disease model, the pathology occurs naturally. It can be a spontaneous mutation that transmits to the next generation or it can be a pathology that develops due to environmental conditions. Animals, strains, or breeds develop pathologies without any human intervention. When a disorder that is previously not described in humans occurs in humans, and when this disorder occurs naturally in a non-human species (for instance bovine spongiform encephalopathy), such animals are termed “orphan disease models”. Negative disease models refer to species or strains in which a certain disease does not develop. These models can be used to study the absence of a disease phenotype, providing insights into the protective mechanisms or roles of specific genes in disease development [1,2].

Nevertheless, this classification may serve only as a guide, as animal disease models can be induced by a combination of two or more of the methods stated above.

The choice of animal model is a careful and clearly defined process. While animals may respond similarly to humans from a physiological or pathological perspective, there may be significant species-by-species or strain-by-strain differences in the underlying mechanisms. For instance, morphine, an efficient but addictive painkiller in humans and C57BL/6J mice, is ineffective and nonaddictive in DBA/2J mice [34].

An ideal or universal animal disease model does not exist. There is an ongoing tendency in research to identify or create better models. This is a consequence of constant progress in the understanding of human diseases. Interestingly, although the use of animal models helps decipher mechanisms of human pathology, the increasing knowledge of the underlying mechanisms and intricate interplay of factors contributing to human diseases forces the constant progress and improvement of the models. The models need to meet the needs of science in reality.

Advancements in science and technology have opened up new avenues for characterizing models. Research in animal studies has shifted toward a deeper investigation of the underlying molecular mechanisms of disease. This evolution in knowledge underscores the necessity for a critical re-evaluation of the current models, a vital process in scientific research.

**What Does This Collection Offer?**

The collection of articles delves into a wide range of animal disease models.

This collection contains review articles on the following topics:

- The opportunities, hurdles, and challenges in perinatal asphyxia pathophysiology in human and animal models [35].
- The underlying mechanisms of monoamine neurotransmitters in the brains of human patients and animals with autism spectrum disorder (ASD) after exposure to valproic acid (medicine for treatment of epilepsy, migraine, and mood disorders). Valproic acid-induced animal models of ASD [36].
- The risk factors in early life/pregnancy associated with hypertension in human offspring and animal models of hypertension with developmental origins; the underlying mechanisms and issues to consider when selecting a model [37].
- The opportunities, limitations, and challenges in mouse studies of cisplatin toxicity [38].
- The use of pigs in testing cardioplegic solutions for cardiopulmonary bypass surgery in humans, their comparability and similarities in heart anatomy and physiology, and current challenges in this area [39].
- A systematic review of the therapeutic approaches used in animal models of interstitial cystitis/urinary bladder pain syndrome [40].

This collection also includes the following research articles on the development of new animal models:

- Using bats as a model of brain aging and neurodegeneration due to their similarities with human hippocampal formation anatomy [41].
- A mouse model of CXCR4-transduced endometrial cancer cells to study novel CXCR4-targeted therapies for unresponsive advanced endometrial cancer [42].
- A mouse model of perinatal brain injury that shows a pattern of brain injury which mirrors multiple key aspects of contemporary diffuse human perinatal white matter injury.
- A study presenting the design and characterization of a subcutaneous implant-associated infection model in mice for the early-stage testing of antimicrobial biomaterials.
- A surgically induced rat model of acute intracerebral hematoma to study human acute disorders of cerebral circulation.
- An in vitro model of acute liver failure isolated pig liver with the use of perfusion technologies, originally intended for preservation before transplantation.
- A terrestrial gastropod, Limacus flavus, known as “Yellow slug”, used as a promising model for mucosal irritation studies (Slug Mucosal Irritation assay) [43].

This collection also includes research articles on the further characterization or refinement of the following existing models:

- A BTBR mouse model of idiopathic autism, with mice showing autism-like symptoms (reduced social interaction and play, low exploratory behaviors, high anxiety, unusual vocalization). The study investigated the underlying mechanisms (gene and protein expression) in BTBR mice and compared it to human ASD and schizophrenia patients to elucidate brain region-specific molecular abnormalities in BTBR mice and their relevance to abnormalities seen in human patients. The results show that the underlying mechanisms between mice and humans differ. Similarities are found only in a small number of genes, which raises concern [44].
- C57BL/6J-*Pitx2^egl1^*/Boc mouse, a genetically modified model for early-onset glaucoma. The mice developed elevated intraocular pressure by four weeks of age, which subsequently became more severe. The study presents the protocols and results regarding the anterior segment morphology, aqueous humor outflow facility, intraocular pressure elevation, and retinal ganglion cell and optic nerve head degeneration in mice from 3 weeks to 12 months of age.
- Tau-P301L mouse, a model of tauopathies (neurodegenerative disease). The study exposes sex-related differences [45].
- A dexamethasone-induced rat model of iatrogenic chronic hypercortisolism. The study presents a refined protocol that triggers adipose tissue redistribution and metabolic changes, characteristics similar to human hypercortisolism/Cushing’s syndrome.
- Mouse model of bariatric surgery. In standardized conditions, the study evaluates orosensory perception (taste, licking behavior) of energy-dense nutrients (oily and sweet stimuli) in mice after bariatric surgery, comparing the two methods.
- Diffuse midline glioma patient-derived xenograft mouse model. The study demonstrates a refined protocol to improve the standardization of, and resemblance with, human malignancy progression for therapeutic testing.

This collection also includes research articles on the testing safety of, and therapeutic agents in, the following existing models:

- Inbred mice. The study shows that short-term exposure to electromagnetic fields in cardiac cells and tissues did not change apoptotic cell death or the expression of the myocardial antioxidant defense system.
- Taxol-induced mouse model of peripheral neuropathy. The study shows that gabapentin protects against induced pain.
- Non-small-cell lung cancer mouse model, a tumor cell transplantation model. The study shows that a low dose of metformin did not affect tumor growth.
- Using pigs as a model to study skin healing. The study excellently demonstrates a protocol for, and provides an in-depth analysis of, the quality of restored tissue down to the molecular level [46].

In an era of rapid scientific advancements, it is crucial to continually revisit and refine our understanding and use of animal models. While the behavioral, clinical, or morphological features of an animal model may exhibit similarities to those of human diseases, the underlying biological mechanisms are not always fully elucidated. Using such models enhances the risk of overlooking species-specific or model-specific differences, which could lead to misunderstandings, misinterpretations, or erroneous conclusions.

A carefully planned animal study necessitates a deep comprehension of the similarities and differences in responses between humans and animals, integrating this knowledge into the study’s objectives. Trust in the relevance of results obtained using an animal model to human disease can only be established if the relationship between the model and the human condition is clearly understood.

As editors, we thank the authors for their insightful contributions and the reviewers for their rigorous evaluation. We hope that the findings presented in this collection will inspire further research and innovation in the field of animal models of human pathology.

We invite readers to explore the diverse range of articles in this collection and engage with the exciting developments and challenges currently ongoing in biomedical research. Together, let us continue to push the boundaries of knowledge and strive for better health outcomes for all.

Thank you for your interest and support.
